# Case report: Clinical fungal endocarditis as a component of presumptive systemic aspergillosis infection in a dog

**DOI:** 10.3389/fvets.2025.1471231

**Published:** 2025-03-04

**Authors:** L. Kay Drake

**Affiliations:** Veterinary Specialty Hospital - North County, Internal Medicine, Ethos Veterinary Health, San Marcos, CA, United States

**Keywords:** aspergillosis, aspergillus, fungal, dog, endocarditis

## Abstract

A 2-year-old 26-kilogram female spayed mixed breed dog presented for acute azotemia, a new heart murmur, anorexia, and lethargy. Further workup revealed marked azotemia with bilateral renal changes on abdominal ultrasound. An echocardiogram detected vegetative lesion on the mitral valve, consistent with infective endocarditis. The patient tested positive for the galactomannan antigen (Aspergillus EIA Galactomannan Test by MiraVista) leading to a presumptive diagnosis of systemic aspergillosis. Despite aggressive treatment, the patient continued to deteriorate and developed signs of dyspnea which temporarily improved with furosemide therapy (VetOne; Boise, ID). After humane euthanasia, postmortem evaluation confirmed fungal endocarditis. This is the first published case of clinical endocarditis in a dog suspected to be from aspergillosis based on both antemortem diagnostics and postmortem pathology. Thus, this case serves to further our understanding of systemic aspergillosis manifestations which in turn can aid in prompt diagnosis and treatment of the condition.

## Case

A 2-year-old 26-kilogram female spayed mixed breed dog presented to Veterinary Specialty Hospital (VSH) for lethargy, acute severe azotemia, and a new grade IV/VI left apical systolic heart murmur. She was originally adopted at 6 months of age and has no reported previous clinical concerns aside from presumed urinary sphincter mechanism incontinence which was controlled with phenylpropanolamine hydrochloride (Proin; PRN Pharmacal; Pensacola, FL). The patient had hematology and biochemistry values within reference intervals 2 months prior to presentation. Two weeks before presentation the patient had seen her primary veterinarian for decreased activity and appetite. She transiently improved following a course of non-steroidal anti-inflammatories which were prescribed to address suspected cervical pain. Due to continued malaise the primary veterinarian performed lab work which revealed polycythemia (72% hematocrit), marked azotemia (BUN 83 mg/dL, creatinine 3.9 mg/dL), hyperphosphatemia (8.3 mg/dL), hyperglobulinemia (5.4 g/dL), isosthenuria (1.010), and a suspicion for bacteriuria. Upon presentation to VSH the patient was estimated to be 5% dehydrated; an oscillometric blood pressure was normal and a WITNESS Lepto Rapid Test (Zoetis; Kalamazoo, MI) was negative. The patient was admitted on intravenous fluids (LRS IV 90 mL/h), ampicillin sodium/sulbactam sodium (780 mg IV q 8 h; Unasyn; Pfizer; New York, New York), maropitant citrate (26 mg IV q 24 h; Cerenia; Zoetis; Kalamazoo, MI) and pantoprazole sodium (26 mg IV q 12 h; Protonix; Pfizer; Philadelphia, PA).

**Figure 1:**
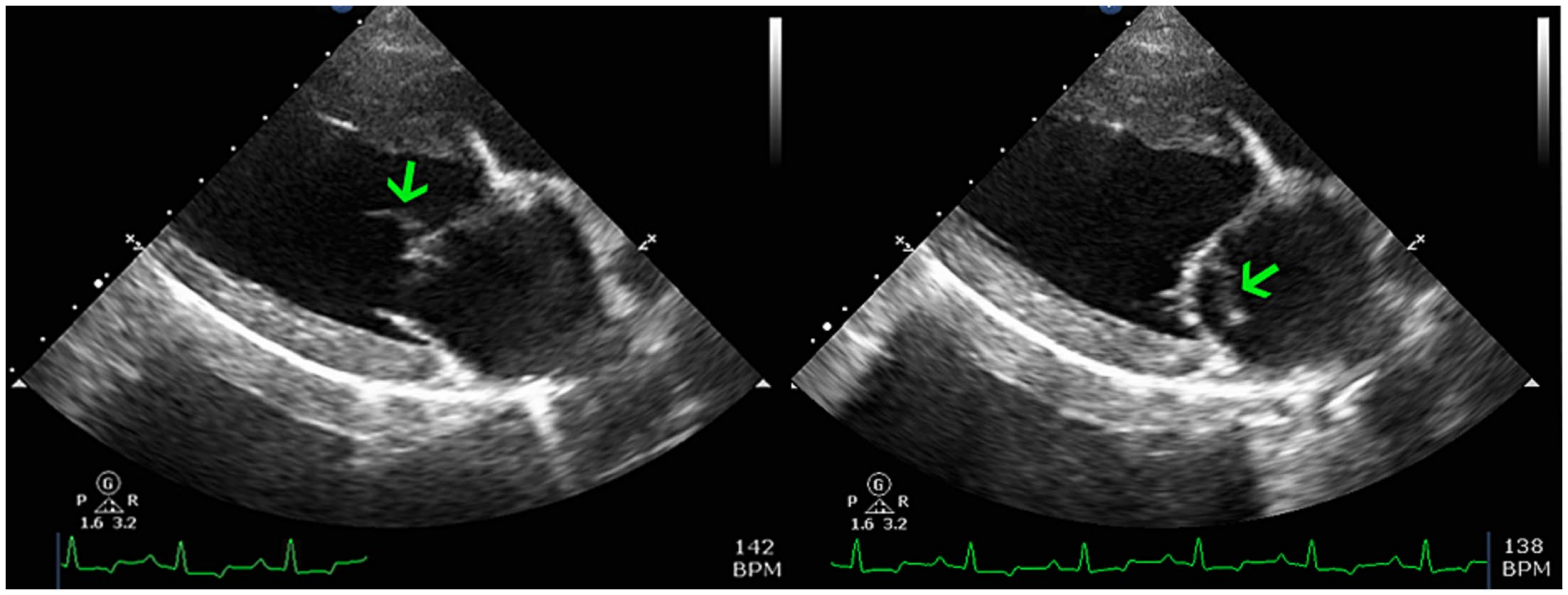
Two dimensional right parasternal long-axis echocardiogram views highlighting the left atrium, left ventricle, and mitral valve apparatus. There is an oscillating vegetative lesion on the anterior leaflet (green arrows) which is most consistent with infective endocarditis.

The following morning the patient transferred to the Internal Medicine service. An abdominal ultrasound detected severe bilateral pyelectasia with proximal ureteral dilatation but a normal urinary bladder. A urine sample had already been collected by the primary veterinarian and a urine culture was later reported to be negative, although the method of collection and cytologic findings were not documented. Renal pelvis contents were echogenic. The uterine stump was also subjectively enlarged but not further investigated. Due to the newly auscultated heart murmur an echocardiogram was recommended but declined by the client. The patient was hospitalized for a total of 5 days with daily PCV and chemistry profile panels. The azotemia mildly improved, the creatinine reaching a nadir of 2.9 mg/dL. On day 3 the client approved the echocardiogram with a board-certified cardiologist, which identified a thickened anterior mitral valve leaflet which was also associated with a vegetative lesion (Figure 1). Mild mitral valve insufficiency was also noted but no other valve changes were evident. The left ventricle was normal in size, while the left atrium was mildly enlarged. The LA:Ao was 1.68, the was LVIDd 42.7 mm, and the MV Peak E velocity was 1.44 m/s. Based on these findings, in addition to the previous diagnostic results, the patient was diagnosed with infectious endocarditis. The diagnosis was also in agreement with the modified Duke Criteria in which the patient met 1 major criteria (echocardiogram findings) and two minor criteria (pyrexia, new or worsening heart murmur) (1). A limited abdominal ultrasound was also repeated showing persistently dilated renal pelvises with hyperechoic contents. Bartonella titers and an aspergillosis galactomannan antigen test (Aspergillus EIA Galactomannan Test by MiraVista) were submitted. The concern for possible fungal etiology was due to the patient’s presumed breed mix (suspect German Shepherd dog inclusion), ultrasonographic changes, multiple organ involvement, and persistent hyperglobulinemia (now at 3.9 mg/dL). The patient remained hospitalized until her voluntary appetite began to improve, which occurred after starting cisparide (10 mg/mL; Nordahl Pharmacy; San Marcos, CA) and nasogastric tube feeding on day 4. However, the patient was also becoming clinically overhydrated. Due to a rising respiratory rate thoracic radiographs were created on day 5 and revealed diffuse moderate to severe interstitial pattern with mild pulmonary venous dilation, despite a normal vertebral heart score (VHS) of 9.6 [normal 9.7 ± 0.5 vertebrae (2)] and a vertebral left atrial size (VLAS) of 2.1 (≤2.3) (3). Due to the concern for emerging congestive heart failure (CHF) the patient was empirically started on diuretic therapy (furosemide 50 mg IV q 8 h) and there was improvement in her respiratory rate and effort, though they remained elevated above normal parameters. Pimobendan, nor another inotropic medication was not administered due to the lack of cardiology support on site at the time; there was also concern for exacerbating the patient’s azotemia as a known possible side effect. While a multimodal etiology for dyspnea is likely, the radiographic changes and diuretic responsiveness heightened suspicions for early CHF. Other likely contributing factors or differentials include thromboembolic disease and vasculitis. Due to financial limitations, concern for continued IV fluid intolerance, and the mildly improved appetite as well as azotemia, the patient was discharged on day 5. At the time of discharge the patient was prescribed amoxicillin-clavulanic acid 375 mg PO q 12 h (Clavamox; Kalamazoo, MI), enrofloxacin 272 mg PO q 24 h (Baytril; Elanco; Shawnee Mission, KS), capromorelin 78 mg PO q 24 h PRN (Entyce; Aratana; Leawood, KS), omeprazole 20 mg PO q 12 h (Prilosec OTC; Procter & Gamble; Cincinnati, OH), maropitant citrate 60 mg PO q 24 h PRN (Cerenia; Zoetis; Kalamazoo, MI), cisparide 13 mg PO q 8 h (10 mg/mL; Nordahl Pharmacy; San Marcos, CA), furosemide 50 mg PO q 8 h (VetOne; Boise, ID), and doxycycline hyclate 150 mg PO q 12 h (Viona Pharmaceuticals; Cranford, NJ).

The patient’s condition continued to deteriorate as noted by the primary veterinarian on day 8. She developed more significant dyspnea and hypoxemia (SpO_2_ 93%). A new set of thoracic radiographs revealed mild left atrial enlargement despite a normal VHS (10), mild cranial lobar venous congestion compared to the arteries, and a mild interstitial pulmonary pattern in the left caudal lung lobe. A VLAS was not provided. The radiologist interpretation of the combination of these findings, in conjunction with the clinical history, was concerning for early left-sided CHF. It should be noted that the some of the radiographic changes noted were less severe than the previous study, despite the relapsing dyspnea. This suggests obfuscation from ongoing furosemide (VetOne; Boise, ID) therapy, an alternative etiology such as thromboembolic disease, or a combination of these possibilities as well as vasculitis. That same day the galactomannan antigen test returned strongly positive (8.63; reference interval is <0.5). With a presumptive diagnosis of systemic aspergillosis, antifungal treatment was initiated immediately (itraconazole PO 5 mg/kg q 12 h) but patient response was limited. The patient was euthanized by the primary veterinarian the following day due to declining quality of life and poor long-term prognosis.

**Figure 2:**
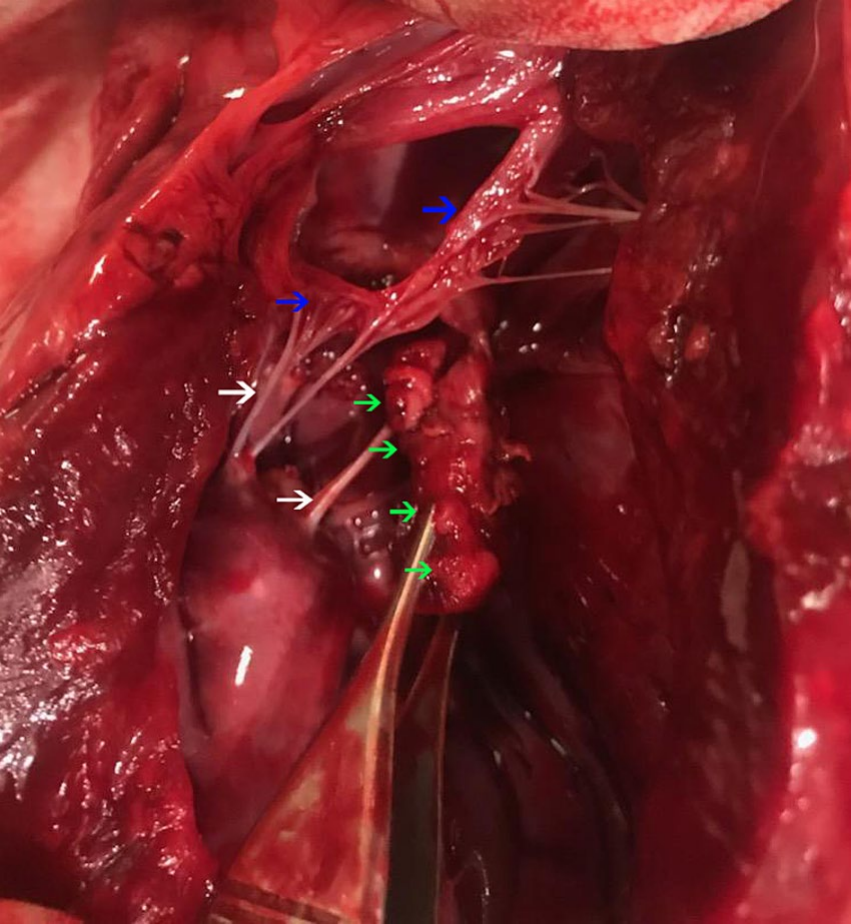
A post-mortem evaluation of the heart. The left ventricular free wall is incised. There is a multi-lobulated vegetative lesion (green arrows) intimately associated with the mitral valve (blue arrows) at the junction with chordae tendinae (white arrows).

**Figure 3:**
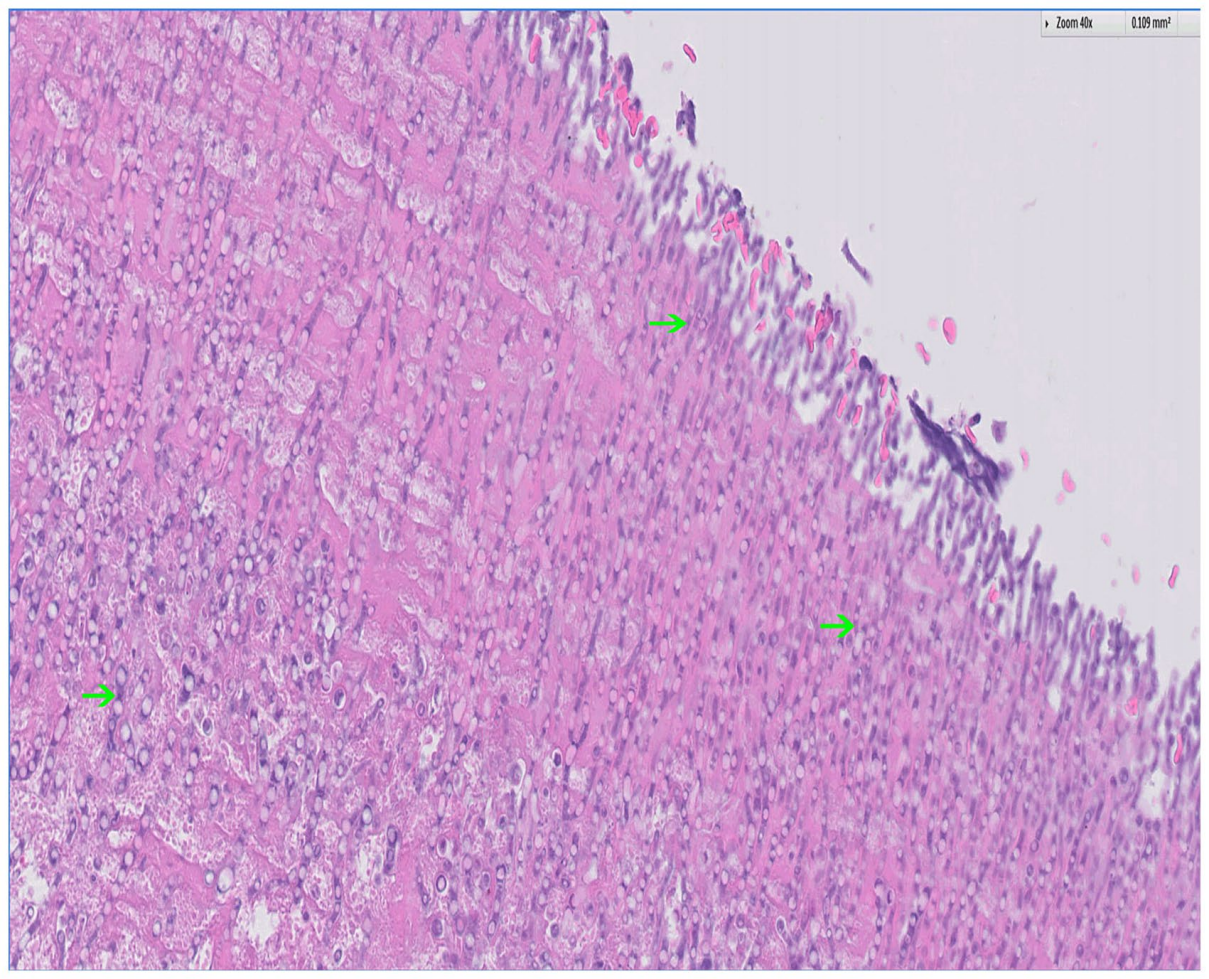
Histopathology of the tricuspid valve with hematoxylin and eosin (H&E) stain; 40x. There is background of fibrin (eosinophilic) with a plethora of fungal hyphae noted throughout (basophilic); several examples are indicated by the arrows. The hyphae are narrow and septate with acute angle branching, a feature consistent with *Aspergillus*. *Courtesy of Sam Jennings, DVM, MSpVM, DACVP of Ethos Diagnostic Science.*

**Figure 4:**
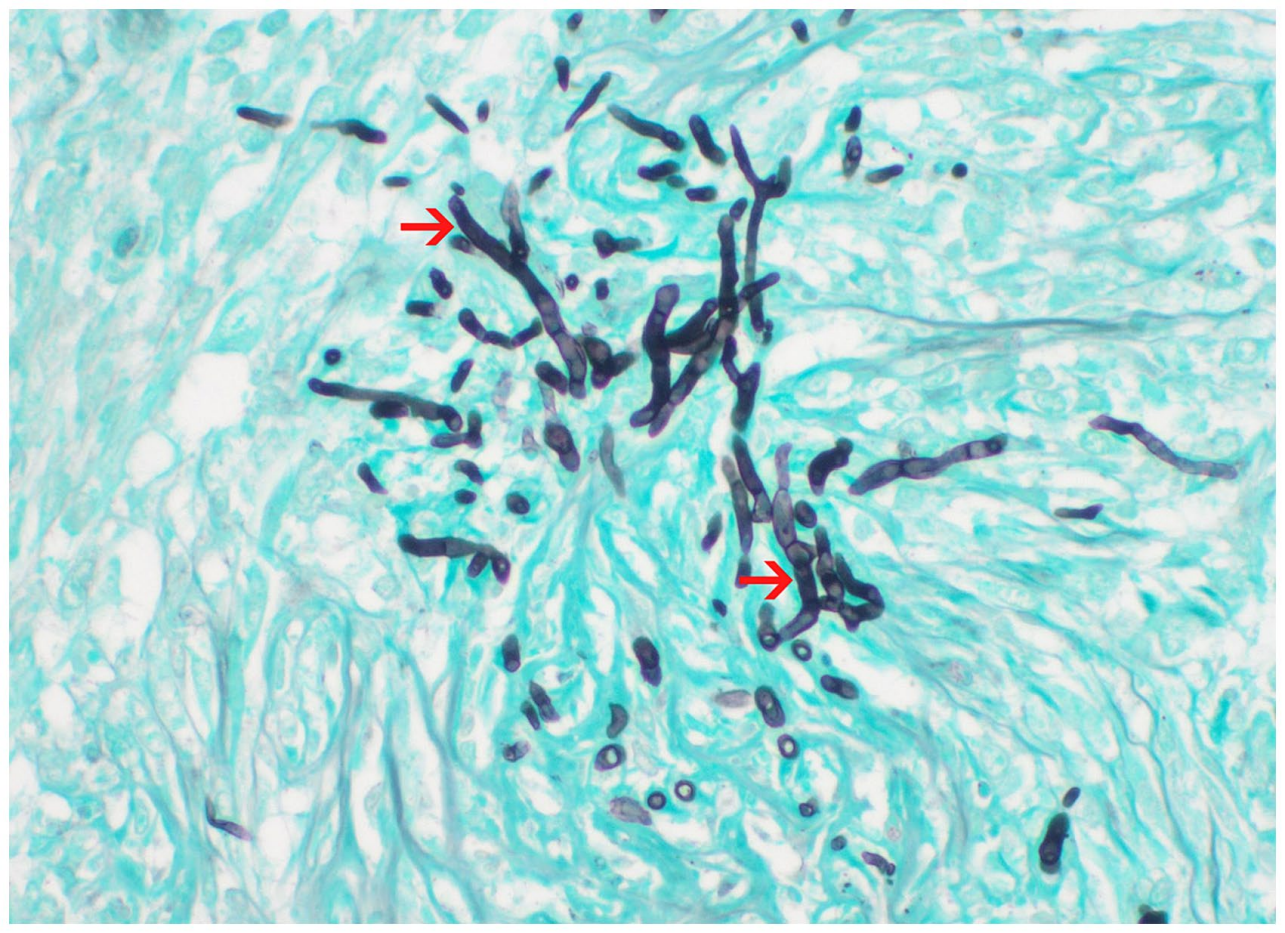
Histopathology of the kidney processed with Grocott-Gömöri’s methenamine silver (GMS) stain; 60x. Fungal hyphae from the center of pyogranulomas within the renal parenchyma. *Courtesy of Aline Rodrigues Hoffmann, DVM, PhD, DCVP, Dermatopathology Specialty Service of Texas A&M University College of Veterinary Medicine & Biomedical Sciences.*

Postmortem evaluation by the referring veterinarian revealed vegetative lesions on both atrioventricular valves (Figure 2). Samples of the heart and left kidney were submitted for histopathology. Disseminated fungal infection was confirmed with hyphae noted in the tricuspid valve, myocardium, renal vessels (Figures 3, 4).

## Discussion

*Aspergillus* is an opportunistic fungal pathogen that is common in the environment. Most clinical infections are limited to fungal rhinosinusitis, caused by *A. fumigatus* infecting immunocompetent dogs (4). Systemic aspergillosis is an uncommon but devastating disease that most commonly results from other *Apergillus* species and typically manifests with renal failure, diskospondylitis, osteomyelitis, and lymphadenomegaly (4). German Shepherd dogs are overrepresented and may have a genetic predisposition due to a hereditary immune defect (5–7). Young to middle-aged females may also be overrepresented (8). While the prognosis is generally poor, treatment with antifungal medication may extend survival for over a year (9). Localized bronchopulmonary aspergillosis may have the best prognosis with possible curative medical treatment, though surgical intervention is also required in some cases (10, 11).

Aspergillosis-induced clinical endocarditis has been well documented in human literature, but the author cannot identify any confirmed cases in veterinary literature. As a general category, fungal endocarditis has been rarely documented in dogs. Fungal myocarditis, and one case of clinical endocarditis, has been reported but the etiologic agent was *Blastomyces* (12). A review of 30 dogs with systemic aspergillosis reported cardiac involvement on some postmortem findings, but the dogs did not demonstrate signs of heart disease aside from one case of ventricular bigeminy (8). Additionally, only one case was classified as endocarditis, the remaining relevant cases were either epicarditis or myocarditis (8). It should be noted that another dog did have suspicion of fungal endocarditis on echocardiogram, but the diagnosis was not confirmed by postmortem examination (8). The subject of this report may represent the first presumptive case of *Aspergillus*-induced endocarditis with clinical manifestation of disease, including suspected heart failure, reported in a dog.

Fungal endocarditis has been poorly characterized in veterinary literature. Literature in human medicine describe a generally poor prognosis, with a mortality rate around 60% (13). The most common human fungal pathogens are *Candida* (46.6–50% of cases) and *Aspergillus* (24–25% of cases) (14, 15). Age varies with the etiology of infection; *Candida* is more common in younger patients, especially neonates relying on indwelling venous catheters fore parenteral nutrition (14). *Aspergillus* becomes more common as age increases (14). There are many proposed risk factors for fungal endocarditis in humans (14, 15). In this author’s experience the most pertinent risk factor to opportunistic fungal infections in veterinary medicine is immunosuppression. This has also been supported by literature focused on opportunistic cutaneous fungal infections for dogs receiving chronic immunomodulating medication (16). Other presumed pertinent risk factors in veterinary medicine include to underlying cardiac abnormalities, indwelling central venous catheters, prolonged antibiotic use, and cardiovascular surgery (15).

Fungal endocarditis has highly variable manifestations, regardless of species. Of the many possible manifestations, pyrexia is a common feature of fundal endocarditis in humans, yet, despite this, over 50% of human patients with *Aspergillus* endocarditis had negative blood cultures (14, 15). Preferred treatment for human aspergillosis endocarditis is multimodal, often utilizing two antifungal agents along with surgical debridement of vegetative lesions (15). When treated with only medical therapy, a mortality rate up to 100% has been reported (15). Therefore, the proposed standard of care in human medicine includes aggressive and early surgical intervention (15, 17). While antifungal therapy has been previously cost prohibitive in veterinary medicine, many of the preferred options of human therapy are becoming more affordable for our canine patients. *Aspergillus* endocarditis in humans is commonly treated with voriconazole (Pfizer; NY, NY) and/or amphotericin B lipid complex injection (Abelcet; sigma-tau pharmaceuticals, Inc.; Gaithersburg, MD), but itraconazole (Sporanox; Janssen Pharmaceuticals, Inc.; Titusville, NJ) and caspofungin (CANCIDAS; Merck Sharp & Dohme B. V.; Waarderweg 39, The Netherlands) are also used in refractory cases (15). Other therapies include flucytosine (Ancobon; Valeant Pharmaceuticals International, Inc.; Bridgewater, NJ) and micafungin (MYCAMINE; Astellas Pharma US, Inc.; Northbrook, IL) (15). Unfortunately, despite aggressive treatment the prognosis is still grave for fungal endocarditis in people, with a survival rate of 32% associated with multimodal therapy and 4% with antifungal therapy alone (15). Additionally, *Aspergillus* endocarditis has been associated with embolic sequelae (including myocardial infarction), pericarditis, and ruptured chordae tendineae (14, 15). Given the severity of the condition, it has been suggested that empirical use of amphotericin B in humans should be considered for immunosuppressed patients with a persistent fever and lack of response to antibiotic therapy (15).

Infective endocarditis in veterinary literature has been mostly linked to bacterial infections and typically manifests with acute congestive heart failure and/or immune-mediated disease (e.g., glomerulonephritis or immune-mediated polyarthritis) (18). The most common pathogens previously documented in veterinary infective endocarditis are *Staphylococcus* spp., *Streptococcus* spp., and *Escherichia coli* (18). *Bartonella* is noted to be the most common cause of culture-negative infective endocarditis (18). Given the emerging and growing evidence that fungal organisms can infiltrate cardiac tissue, the author proposes fungal etiologies be considered during the diagnostic workup process for infective endocarditis. Currently, diagnosis relies on echocardiographic findings in conjunction with clinical manifestation of signs. Human medicine utilizes the Modified Duke scoring system to aid in diagnosis and a veterinary equivalent has been proposed (19). However, this veterinary model still prioritizes a bacterial etiology with reliance, in part, on blood cultures (19). Including fungal testing, such as antigen assays or serologic testing, may help better identify cases of fungal endocarditis that have previously been unrecognized. In spite of ongoing efforts to improve the diagnostic approach, a significant proportion of patients with fungal endocarditis are diagnosed post-mortem (10–33% reported in humans) (13, 14). For systemic aspergillosis the Galactomannan antigen assay (ELISA) may be the most helpful diagnostic, but there is potential for cross-reactivity with other systemic mycoses or false-positive results with PlasmaLyte (Baxter Healthcare Corporation; Deerfield, IL) administration (20). Urine antigen assays (ELISA) are also a valuable diagnostic method for *Blastomyces* and *Histoplasma* detection (21, 22). While antibody serology testing may be useful in some cases, the sensitivity and specificity vary (21, 22). Urine cytology and culture can also detect *Aspergillus* infection, but are not considered sensitive tests (23, 24). For most cases of fungal endocarditis histopathology and other forms of cytology or culture are considered impractical.

The case provides valuable insight into the manifestation of systemic fungal disease, however, is limited by numerous logistical factors. Unfortunately, the institution did not have seven-day radiology and cardiology support to help interpret diagnostics and guide treatment options in real-time. Additionally, the post-mortem samples were collected by the referring veterinarian and a full necropsy was not performed. Lastly, speciation of the fungal organism would have been ideal to confirm the diagnosis but when attempted on stored post-mortem samples the DNA was not of sufficient quality to provide definitive results.

Fungal endocarditis remains a poorly characterized condition in veterinary medicine. This case of suspect systemic aspergillosis represents a novel example of clinical *Aspergillus-*endocarditis manifestation. Currently, systemic aspergillosis has a poor-to-grave prognosis. As we continue to better understand the pathophysiology of infection, we may be able to develop earlier detection methods and more effective treatment modalities.

## Data Availability

The original contributions presented in the study are included in the article/supplementary material, further inquiries can be directed to the corresponding author.
